# Application of multi-omics in systemic autoimmune rheumatic diseases: a bibliometric and visualization analysis

**DOI:** 10.3389/fimmu.2026.1759610

**Published:** 2026-04-16

**Authors:** Lili Cheng, Zhongfu Tang, Ming Li, Chuanbing Huang

**Affiliations:** Department of Rheumatology and Immunology, The First Affiliated Hospital of Anhui University of Chinese Medicine, Hefei, Anhui, China

**Keywords:** bibliometrics, genomics, metabolomics, multi-omics, proteomics, single-cell sequencing, systemic autoimmune rheumatic diseases

## Abstract

**Background:**

Multi-omics technologies have increasingly been applied to systemic autoimmune rheumatic diseases (SARDs), yet the global research landscape and thematic evolution of this field remain unclear.

**Objective:**

This study intends to systematically review the application status and development trends of multi-omics technologies in SARDs research over the past two decades via bibliometric analysis, so as to guide future research directions.

**Methods:**

Relevant English literatures on multi-omics application in SARDs were retrieved from the Web of Science Core Collection (WoSCC) database, covering the period from January 1, 2005, to Dec 31, 2025. Multi-omics was operationally defined as studies reporting integration of at least two omics layers in one investigation. After deduplication and visual screening, A total of 2576 documents (2072 research articles and 504 reviews) were included. Bibliometric and visual analyses were performed using CiteSpace, VOSviewer, Phthon and bibliometrix. R package.A complementary PubMed analysis was performed to validate thematic robustness and assess clinically oriented studies.

**Results:**

Annual output increased from 11 publications in 2005 to 525 by 2025, with three developmental phases: exploratory (2005–2015), acceleration (2016–2020), and expansion (2021–2025). China (27%) and the United States (17.3%) accounting for 44.3% of total output. European countries demonstrated higher international collaboration rates (MCP% up to 39.2%). Core institutions included Harvard University and the University of California et al. Keyword and burst analyses indicated a thematic shift from proteomics and synovial fluid profiling to epigenetics and metabolomics, and most recently to single-cell RNA sequencing–driven immune cell differentiation research. Citation analysis revealed a centralized intellectual structure anchored in high-impact immunology and rheumatology journals. PubMed validation confirmed consistent growth patterns and thematic concentration on biomarker discovery and mechanistic studies.

**Conclusion:**

Multi-omics research in SARDs has progressed from bulk molecular characterization toward high-resolution immune cell–level investigation, with increasing emphasis on biomarker stratification and immune heterogeneity. By delineating developmental phases, global collaboration patterns, and thematic immunological shifts, this study provides a quantitative framework for evaluating the current maturity and future translational direction of multi-omics research in autoimmune diseases.

## 1 Introduction

Systemic Autoimmune Rheumatic Diseases (SARDs) are a group of heterogeneous diseases characterized by abnormal activation of the immune system, production of autoantibodies and/or autoreactive lymphocytes, leading to systemic multi-organ chronic inflammation and tissue damage. They include rheumatoid arthritis, systemic lupus erythematosus, Sjögren’s syndrome, and other types (1). These diseases not only exhibit high heterogeneity but also involve the interaction of multiple factors such as genetics, immunity, and environment in their pathogenesis, resulting in difficulties in early diagnosis, large differences in treatment response, and inaccurate prognosis assessment (2). Although in recent years, with the development of molecular biology technologies, the academic community has gained a deeper understanding of the pathophysiological processes of SARDs, the current research on the complex regulatory network of these diseases still cannot meet the clinical needs for precise diagnosis and treatment (3).

The rise of multi-omics provides a new idea to break through this research bottleneck. By integrating biological data from multiple dimensions such as genomics, transcriptomics, proteomics, metabolomics, microbiomics, and single-cell sequencing, this technology can systematically reveal the pathogenesis, key regulatory targets, and biomarkers of SARDs at the molecular level. For example, screening disease-susceptible genes through genomics, analyzing the regulatory pathways of differentially expressed genes in combination with transcriptomics, and verifying the expression changes of key molecules using proteomics and metabolomics can construct a more complete map of disease molecular mechanisms, providing a scientific basis for early diagnosis, optimization of treatment regimens, and prognosis monitoring of diseases (4). However, with the continuous accumulation of research results of multi-omics in the field of SARDs, the number of related literatures has increased exponentially.

Several quantitative approaches (e.g., systematic reviews, meta-analyses, scoping reviews, evidence mapping, and text mining) can support field-level synthesis, but they often target narrow questions and may miss large-scale collaboration and citation patterns. Bibliometric analysis, by contrast, can quantitatively map publication trends, collaboration networks, intellectual structure, and thematic evolution across large datasets (5). Because this study focuses on global research architecture and developmental trajectories of multi-omics research in SARDs rather than specific interventions, bibliometric analysis is the most suitable framework. To date, no comprehensive bibliometric study has systematically assessed the field’s development and structural dynamics, leaving a gap in macro-level understanding (6).

**Figure 1:**
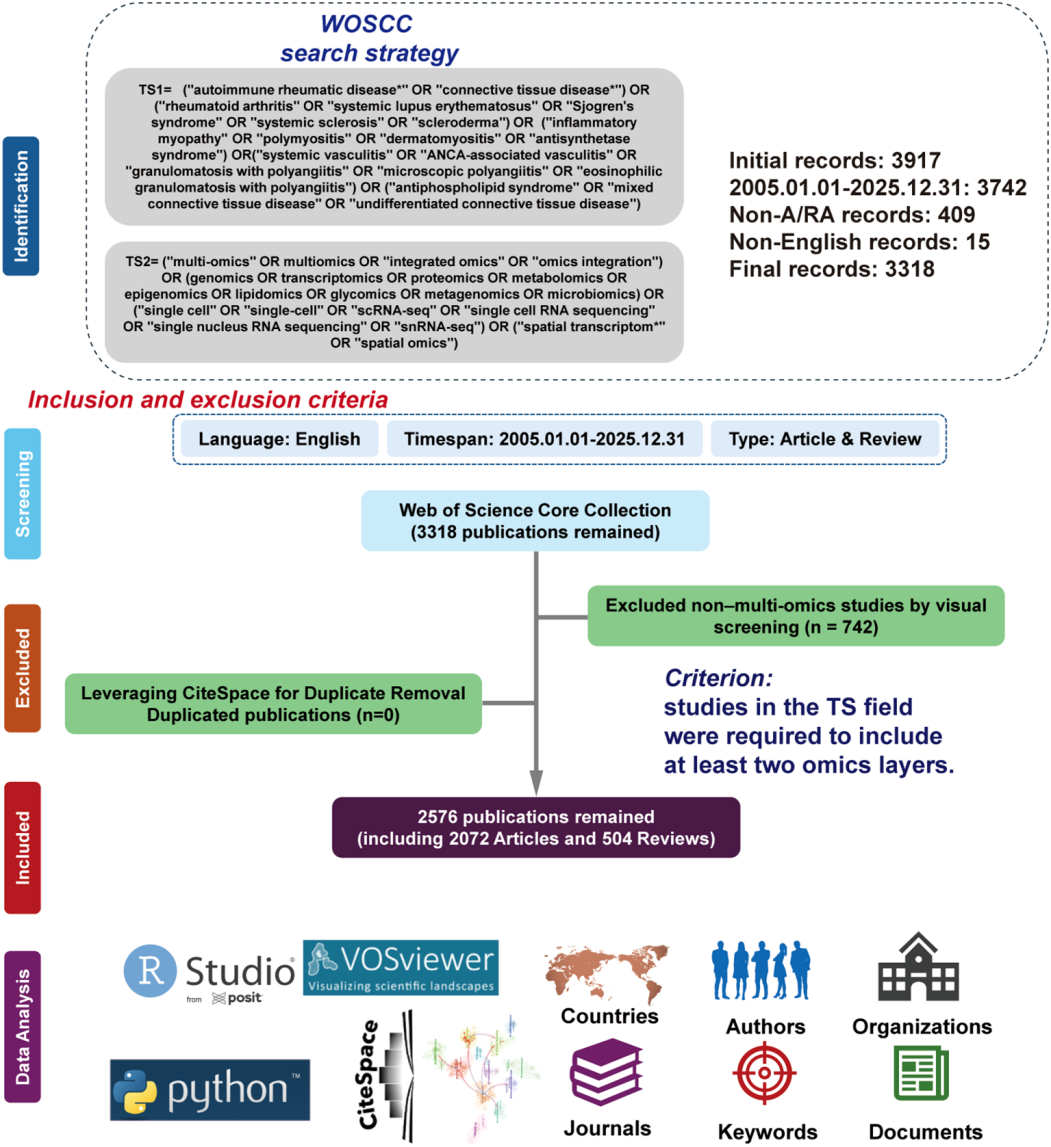
Research framework. WoSCC records (2005–2025) were screened using an operational multi-omics definition (integration of ≥2 omics layers), yielding 2,576 eligible publications. Data were cleaned and standardized, then analyzed for trends, collaboration networks, co-citation structures, and keyword evolution using bibliometrix, VOSviewer, and CiteSpace.

Based on this, this study used bibliometric analysis tools to conduct a systematic research on the application of multi-omics in SARDs The specific research contents are as follows (**Figure 1**): (1) Comprehensively sort out the global literature publication trends, regional distribution characteristics, and research cooperation networks in this field to clarify the global research pattern; (2) Identify high-productivity research institutions, core journals, highly cited authors, and landmark research literatures in the field to clarify the distribution of academic influence in the field; (3) Visualize the evolutionary trajectory of research topics and keyword clustering characteristics in this field in the form of a timeline to explore the change rules of research hotspots; (4) Systematically analyze the application status and achievements of multi-omics technologies in SARDs; (5) Summarize the current technical bottlenecks, data integration problems, and clinical translation obstacles faced by research, and prospect the future research directions, so as to provide references for promoting the in-depth application of multi-omics technologies in the field of SARDs.

## 2 Methods

### 2.1 Data source and search strategy

This study selected the Web of Science Core Collection (WoSCC) as the main data source. WoSCC is one of the most authoritative and widely used databases in bibliometric research, with high-quality literature collection, complete citation indexes, and standardized data formats (7). The search term strategy in this study was developed by integrating bibliometric studies related to multi-omics and SARDs **Supplementary Table 1** provides the search terms used in this study. The retrieval was conducted on Feb 14, 2026, and the search terms were limited to English articles and reviews published from Jan 1–2005 to the Dec31 2025.

In this study, multi-omics research was operationally defined as investigations that integrate data from at least two distinct omics layers within a single research framework for joint analysis. Omics layers included, but were not limited to, genomics, transcriptomics, proteomics, metabolomics, epigenomics, and single-cell omics. Studies involving only a single omics dataset were excluded, even if the terms “multi-omics” appeared in the title, abstract, or keywords, provided that no actual cross-omics integrative analysis was performed. To minimize the risk of misclassification, a random subset of the retrieved publications was manually reviewed to verify compliance with the predefined multi-omics criteria. To estimate potential misclassification, 20 publications were randomly sampled from the retrieved dataset and independently evaluated for compliance with the integrative multi-omics definition. Among these, 17 (85%) met the strict criterion of integrating ≥2 omics layers within a unified analytical framework, whereas 3 (15%) represented parallel or single-omics applications without true cross-omics integration. This suggests a modest but non-negligible risk of over-retrieval due to terminology heterogeneity.

### 2.2 Screening, data cleaning, and reproducible workflow

To reduce the risk of misclassification and enhance data purity, a random sample of the preliminarily screened publications was independently reviewed by three researchers to assess compliance with the predefined multi-omics criteria. A double-blind independent evaluation approach was adopted. In cases of disagreement, a third reviewer adjudicated to reach a final decision (8). The inter-rater agreement among the three reviewers was high (Cohen’s kappa = 0.92; 95% confidence interval: 0.88–0.96), indicating excellent consistency. This procedure effectively minimized classification bias and strengthened the reliability of the screening process.

Prior to formal analysis, systematic preprocessing and disambiguation of the raw bibliographic data were performed. Initial deduplication was conducted using the database’s built-in filtering function, followed by manual verification to ensure complete removal of duplicate records. Subsequently, author names, institutional affiliations, and country information were standardized and disambiguated using the *bibliometrix* package in the R environment. Specific procedures included harmonizing capitalization formats, standardizing abbreviations, merging variant spellings of the same institution, cleaning special characters and empty fields, and applying function-based normalization to reduce network fragmentation caused by inconsistent naming conventions. High-frequency author and institutional names were further manually cross-checked to ensure accurate merging. In addition, key fields (authors, affiliations, publication year, and keywords) were examined for completeness and consistency. All data cleaning, disambiguation, and analytical procedures were conducted within a reproducible and standardized script-based workflow to enhance transparency and methodological reproducibility.

### 2.3 PubMed search for clinically oriented studies

In addition, to validate the robustness of the WoSCC-based findings and to further assess translational orientation, a complementary search was conducted in the PubMed database. Unlike WoSCC, PubMed provides more detailed indexing of clinical study types and trial designs, enabling refined identification of clinically oriented research. This broader PubMed dataset was used to independently examine annual publication trends, thematic structures, and geographic distribution patterns for cross-database validation (9, 10).

Subsequently, to specifically evaluate translational progress at the interventional level, a subset of studies indexed under “Randomized Controlled Trial” was extracted from the PubMed dataset. Eligible randomized controlled trials (RCTs) met the following criteria:(1) indexed in PubMed as “Randomized Controlled Trial”;(2) conducted in clinical patients or based on clinical samples;(3) integrated at least two distinct omics datasets within a unified analytical framework;(4) multi-omics integration constituted a central component of study design;(5) directly related to systemic autoimmune rheumatic diseases, with clearly identifiable information on study population, omics platforms, and analytical methods.

### 2.4 Bibliometric analysis

Multiple bibliometric tools were employed to perform a multidimensional quantitative analysis of the included publications, covering key indicators such as geographic distribution, collaboration networks, institutional contributions, journal co-occurrence networks, and citation structures. Descriptive statistical analyses were conducted using the bibliometrix package (version 5.0) in R (version 4.4.5) (11). These analyses included annual publication output, identification of core authors, journal distribution, and citation analysis. Raw network data were also extracted for further processing. In addition, built-in functions of this package were used to generate thematic evolution bubble matrices to visualize shifts in research hotspots over time.

VOSviewer (version 1.6.20) was applied to construct collaboration and co-authorship networksr (12). Network visualization was generated using the Fruchterman–Reingold force-directed layout algorithm. Association strength normalization was used to control for scale effects, and the Walktrap algorithm was applied for community detection.

CiteSpace (version 6.2.R1) was used for citation network analysis, burst detection, and exploration of research frontier evolution (13).The time slicing interval was set to one year. Node selection followed predefined parameters: g-index (k = 25), LRF = 2.5, L/N = 10, LBY = 5, and e = 1.0.

For visualization refinement and unified formatting, selected raw outputs generated by the above software were imported into the Python environment (version 3.14). Data processing and graphical enhancement were performed using the pandas, matplotlib, seaborn, and networkx libraries. The analytical workflow of this study is presented in **Figure 1**.

## 3 Results

### 3.1 Overall publication characteristics and growth trends

**Figure 2:**
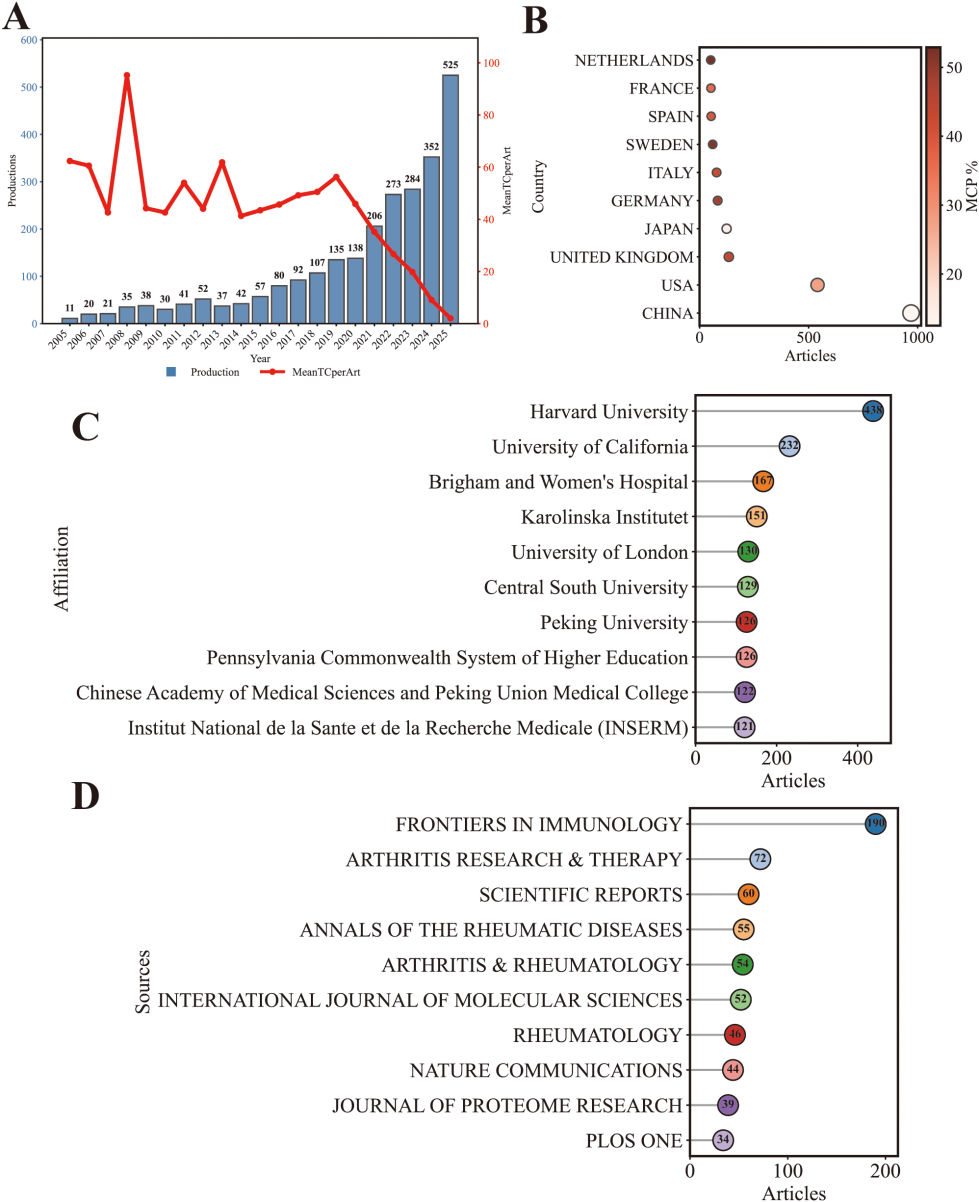
Publication trends and geographic distribution (Based on the WoSCC dataset) **(A)** Annual publication output and annual citation analysis (R package). **(B)** Bubble chart of the top 10 most productive countries (MCP%) (Bubble size represents publication count; MCP% indicates the proportion of multiple-country publications,R package). **(C)** Lollipop chart of the top 10 most productive institutions (R package). **(D)** Lollipop chart of the top 10 most productive journals (R package).

From the WoSCC, a total of 2,576 publications on multi-omics research in (SARDs were retrieved, including 2,072 research articles and 504 review papers. These publications were produced by 17,864 authors from 620 sources and cited 110,493 references, spanning multiple disciplines (**Table 1**). The annual publication output showed a sustained upward trajectory over the study period. From 2005 to 2015, annual publication output in this field grew slowly and remained at a relatively low level overall. Growth accelerated gradually from 2015 to 2020, with the annual article count rising steadily at an increased rate. Post-2020, publication output surged sharply year on year, with growth momentum continuing to strengthen and reaching the highest annual output 525 in the study period by 2025 (**Figure 2A**).

**Table 1:** General characteristics of the database (R package).

Description	Results
Timespan	2005:2025
Sources (Journals, Books, etc)	620
Documents	2576
Annual Growth Rate %	21.32
Document Average Age	5.51
Average citations per doc	27.95
References	110493
Keywords Plus (ID)	5155
Author’s Keywords (DE)	4479
Authors	17864
Authors of single-authored docs	48
Single-authored docs	53
Co-Authors per Doc	9.33
International co-authorships %	26.36
article	2072
review	504

The top 10 countries by publication output were China (993, 27%), the United States (635, 17.3%), Japan (173, 4.7%), Italy (118, 3.2%), France (91, 2.5%), Korea (87, 2.4%), the Netherlands (80, 2.2%), Germany (79, 2.1%), Spain (76, 2.1%), and Sweden (73, 2.0%) (**Table 2**). China and the United States together accounted for 44.3% of total publications, indicating a dominant position in this field. In terms of international collaboration, Germany (39.2%), Spain (36.8%), the Netherlands (33.8%), and Sweden (32.9%) showed relatively high proportions of multiple-country publications (MCP%), whereas China (11.5%) and Korea (11.5%) demonstrated lower MCP percentages. The United States showed a moderate level of international collaboration (19.8%) (**Figure 2B**).

**Table 2:** Top 10 most productive countries in WoSCC and their collaboration rates (R package).

Country	Articles	Articles %	SCP	MCP	MCP %
CHINA	993	27	879	114	11.5
USA	635	17.3	509	126	19.8
JAPAN	173	4.7	153	20	11.6
ITALY	118	3.2	93	25	21.2
FRANCE	91	2.5	64	27	29.7
KOREA	87	2.4	77	10	11.5
NETHERLANDS	80	2.2	53	27	33.8
GERMANY	79	2.1	48	31	39.2
SPAIN	76	2.1	48	28	36.8
SWEDEN	73	2	49	24	32.9

Based on the WoSCC dataset, the top three institutions by publication output were Harvard University (438), University of California (232), and Brigham and Women’s Hospital (167) (**Figure 2C**), the top three journals in terms of publication output were Frontiers in Immunology (190), Arthritis Research & Therapy (72), and Scientific Reports (60) (**Figure 2D**). The leading journals are primarily positioned in immunology and rheumatology.

### 3.2 Research trajectory

**Figure 3:**
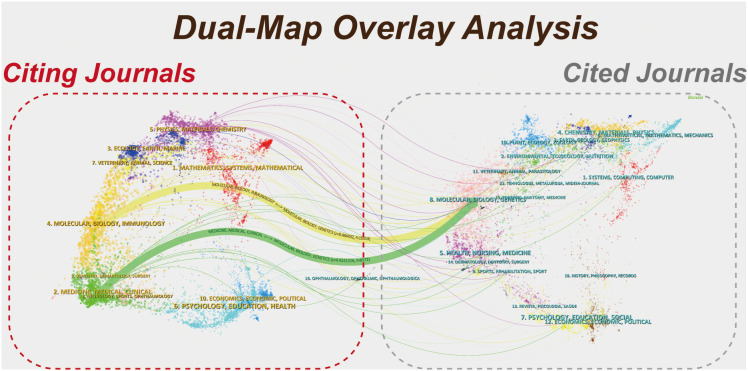
Double-map overlay analysis.(Time slicing: 2005–2025,Citing journals are shown on the left and cited journals on the right;colored trajectories represent major citation paths across disciplinary domains. Line thickness reflects relative citation linkage strength.CiteSpace 6.2.R1).

The dual-map overlay analysis indicates that knowledge diffusion in this field demonstrates a parallel pattern of pronounced interdisciplinarity and internal disciplinary consolidation (**Figure 3**). Three prominent citation pathways are identified. The first pathway extends from Medicine/Clinical to Molecular Biology/Genetics (green trajectory), suggesting that the research is closely linked to clinical practice and disease-oriented investigations, with a strong application-driven orientation. The second pathway runs from Molecular Biology/Immunology to Molecular Biology/Genetics (yellow trajectory), reflecting the continuity and deepening of knowledge within the discipline, as well as the cumulative advancement of theoretical and mechanistic studies. The third pathway originates from Physics/Materials/Chemistry and flows toward Molecular Biology/Genetics (pink trajectory), indicating that the field is substantially supported by methodologies and theoretical frameworks from the physical sciences, thereby exhibiting a notable trend of interdisciplinary integration.

### 3.3 Annual publication output analysis of authors, institutions and journals

**Figure 4:**
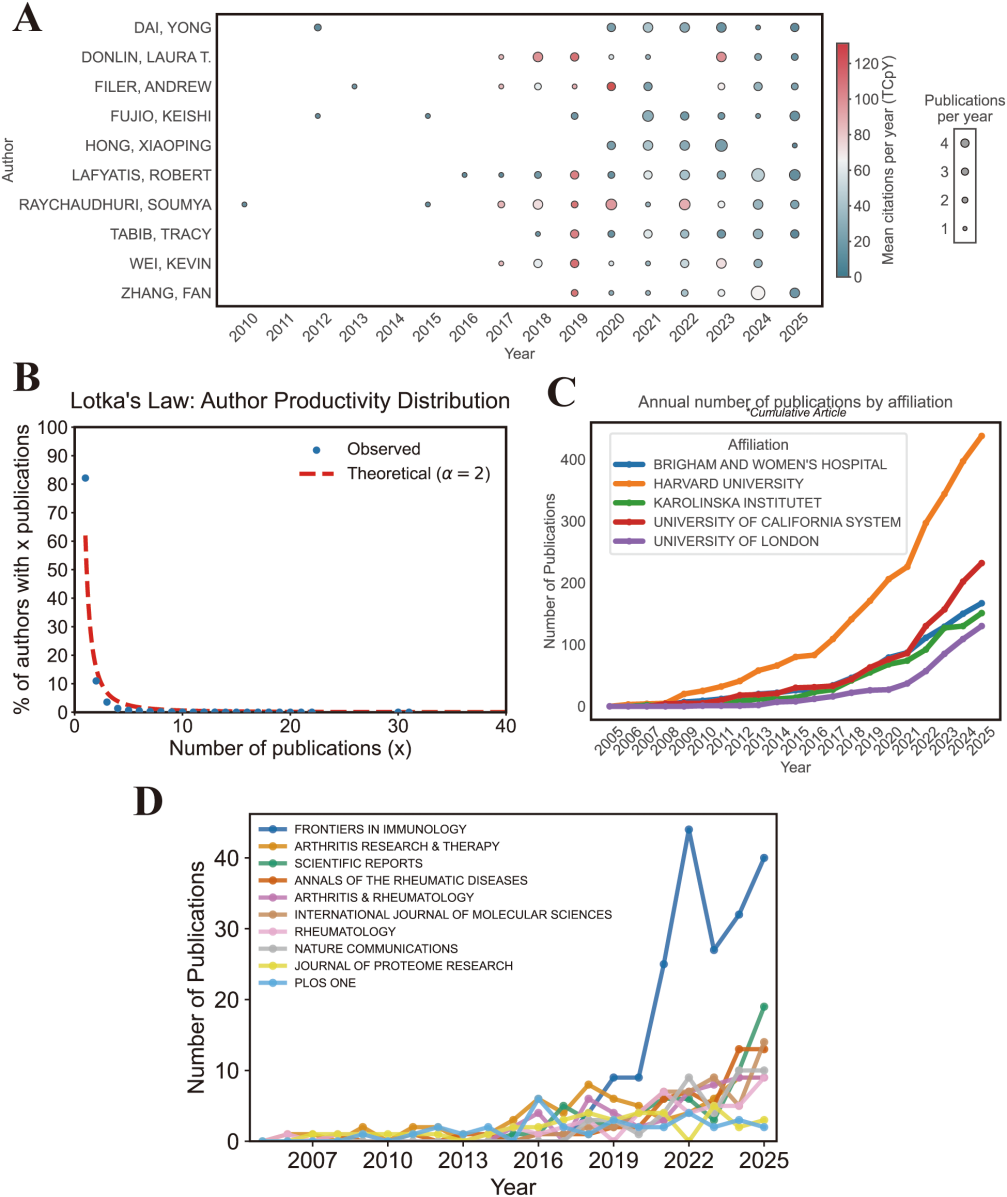
Productivity analysis of authors, institutions, and journals. **(A)** Annual publication output of the top 10 most productive authors (R package). **(B)** Author productivity distribution based on Lotka’s Law (R package). **(C)** Annual publication output of the top 10 most productive institutions (R package). **(D)** Annual publication output of the top 10 most productive journals (R package).

The annual publication output of the top 10 authors increased markedly after 2016 (**Figure 4A**). Raychaudhuri Soumya ranked first with an h-index of 22, a g-index of 30, 30 publications, and 4,328 total citations. Lafyatis Robert (h-index = 19, 31 publications) and Filer Andrew (h-index = 16, 18 publications) also demonstrated strong academic impact. Several leading authors, including Rao Deepak A. and Wei Kevin, showed high total citation counts (>3,000), reflecting substantial influence in this field (**Table 3**). Lotka’s Law analysis showed that most authors published only one paper, while a small proportion of authors produced multiple publications, indicating a highly skewed productivity distribution (**Figure 4B**).

**Table 3:** Top 10 authors ranked by bibliometric indicators (R package).

Author	h_index	g_index	m_index	TC	NP	PY_start
RAYCHAUDHURI SOUMYA	22	30	1.294	4328	30	2010
LAFYATIS ROBERT	19	31	1.727	1829	31	2016
FILER ANDREW	16	18	1.143	3706	18	2013
ROBINSON WILLIAM H.	16	18	0.762	1657	18	2006
RAO DEEPAK A.	15	17	1.5	3217	17	2017
TABIB TRACY	15	22	1.667	1632	22	2018
WEI KEVIN	15	19	1.5	3549	19	2017
DONLIN LAURA T.	14	18	1.4	3780	18	2017
PITZALIS COSTANTINO	14	16	1.273	2052	16	2016
BRENNER MICHAEL B.	13	15	1.444	2715	15	2018

At the institutional level, Harvard University, the University of California, and Brigham and Women’s Hospital ranked among the most productive institutions. Annual output from these institutions increased steadily, particularly after 2018 (**Figure 4C**).

In terms of journals, Frontiers in Immunology ranked first with 190 publications, an h-index of 31, and 3,590 total citations. It was followed by Arthritis Research & Therapy (72 publications) and Annals of the Rheumatic Diseases (55 publications). High-impact journals such as Nature Communications (h-index = 25) also contributed substantially to the field (**Table 4**). The annual output of these journals increased progressively over time (**Figure 4D**), reflecting growing dissemination of multi-omics research in core immunology and rheumatology venues.

**Table 4:** Top 10 journals ranked by bibliometric indicators (R package).

Source	h_index	g_index	m_index	TC	NP	PY_start
FRONTIERS IN IMMUNOLOGY	31	49	3.444	3590	190	2018
ANNALS OF THE RHEUMATIC DISEASES	25	47	1.316	2220	55	2008
ARTHRITIS & RHEUMATOLOGY	25	43	2.083	1950	54	2015
NATURE COMMUNICATIONS	25	44	2.778	3484	44	2018
ARTHRITIS RESEARCH & THERAPY	24	42	1.143	1974	72	2006
INTERNATIONAL JOURNAL OF MOLECULAR SCIENCES	18	26	1.636	756	52	2016
JOURNAL OF PROTEOME RESEARCH	18	36	0.9	1329	39	2007
JCI INSIGHT	17	31	1.545	1047	31	2016
JOURNAL OF AUTOIMMUNITY	17	31	1.133	1112	31	2012
RHEUMATOLOGY	17	28	0.81	869	46	2006

### 3.4 Collaboration networks among authors, countries, and institutions

**Figure 5:**
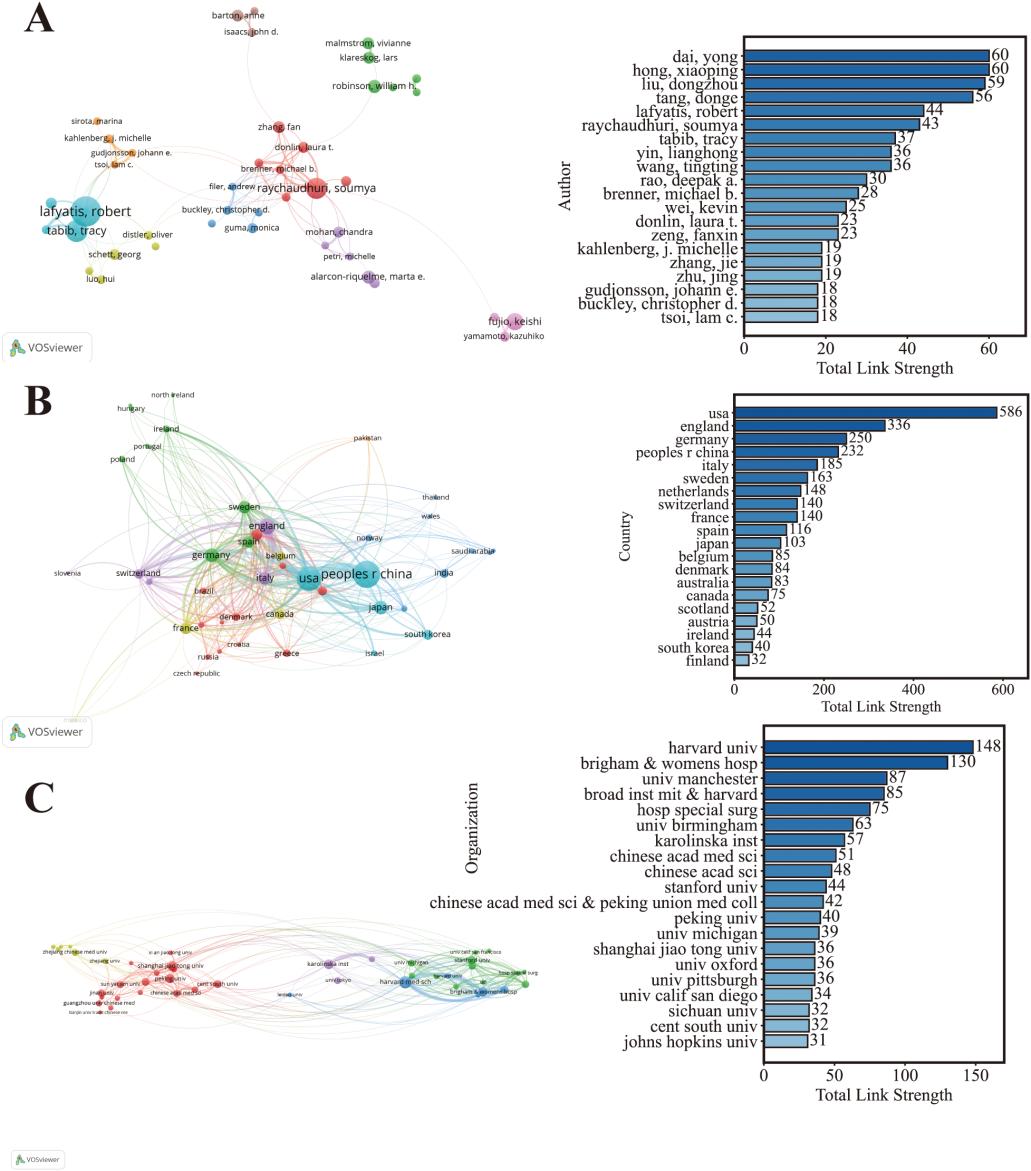
Collaboration networks among authors, countries, and institutions. **(A)** Author collaboration network with total link strength (Nodes represent authors; node size indicates publication output; links represent co-authorship ties; link thickness reflects TLS,publication-count threshold ≥ 5,VOSviewer). **(B)** Country collaboration network with total link strength (Nodes represent countries; node size indicates publication output; links represent international co-authorship; link thickness reflects TLS,publication-count threshold ≥ 30,VOSviewer). **(C)** Institutional collaboration network with total link strength (Nodes represent institutions; node size indicates publication output; links represent inter-institution co-authorship; link thickness reflects TLS,publication-count threshold ≥ 20.Select the top 20 items based on the top 20 by total link strength.VOSviewer).

Cooperation networks based on publication volume were constructed from three dimensions: authors, countries, and research institutions, with Total Link Strength (TLS) as the quantitative indicator to intuitively present the connection tightness, collaboration breadth, and core cooperation pattern of cooperative subjects across all dimensions (**Figure 5**). **Figure 5A** revealed multiple core author collaboration clusters, with Lafyatis, Robert, Raychaudhuri, Soumya, and Donlin, Laura T. as the core node authors who had relatively higher Total Link Strength and served as key hubs in the author cooperation network of this field. Dai, Yong, Hong, Xiaoping, Liu, Dongzhou and other authors achieved the highest TLS values, and close cross-team collaborative connections were formed among authors.

The United States and the People’s Republic of China were the two core nodes in the national cooperation network of this field. In addition, the USA and England had significantly higher TLS than other countries, acting as the core hubs of international scientific research cooperation. Germany, Japan, South Korea and other countries also emerged as important cooperative subjects, forming diverse collaborative connections with core countries and surrounding nations (**Figure 5B**).

**Figure 5C** displayed the collaborative connections and TLS distribution of core research institutions. Top overseas universities and research institutions constituted the core of the cooperation network, among which Harvard univ, Brigham & womens hosp, broad inst MIT & Harvard and other institutions took the lead in TLS and became the core hubs of international institutional cooperation. Karolinska inst, univ Manchester, Stanford univ and other well-known overseas universities also served as important cooperative nodes. Among domestic research institutions, Chinese acad med sci, Peking Union Medical College, Peking univ, shanghai jiao tong univ and others ranked among the core cooperation circles and formed close collaborations with top overseas institutions and domestic counterparts. In the overall institutional cooperation network, top domestic and overseas universities/medical institutions had close cross-institutional and cross-national collaborative connections, forming a multi-level cooperation system with core institutions as hubs and radiating to numerous universities and research institutes.

### 3.5 Research hotspots and frontier evolution based on keywords

#### 3.5.1 High-frequency keywords

The top 20 high-frequency keywords are presented in **Table 5**. The most frequent keywords were rheumatoid arthritis (820), systemic lupus erythematosus (490), and expression (463), followed by activation (200), inflammation (195), and identification (180). These results indicate that rheumatoid arthritis represents the most intensively studied disease within SARDs, and that gene expression profiling and biomarker identification constitute major analytical focuses in multi-omics research.

**Table 5:** Top 20 most frequent keywords (CiteSpace).

Keywords	Count
rheumatoid arthritis	820
systemic lupus erythematosus	490
expression	463
disease	261
activation	200
rheumatoid-arthritis	198
inflammation	195
identification	180
pathogenesis	158
association	141
classification	140
antibody	117
systemic sclerosis	117
biomarkers	112
autoantibody	108
risk	105
mass spectrometry	100
mechanisms	96
gene expression	93
sjogrens syndrome	88

#### 3.5.2 Keywords clusters

**Figure 6:**
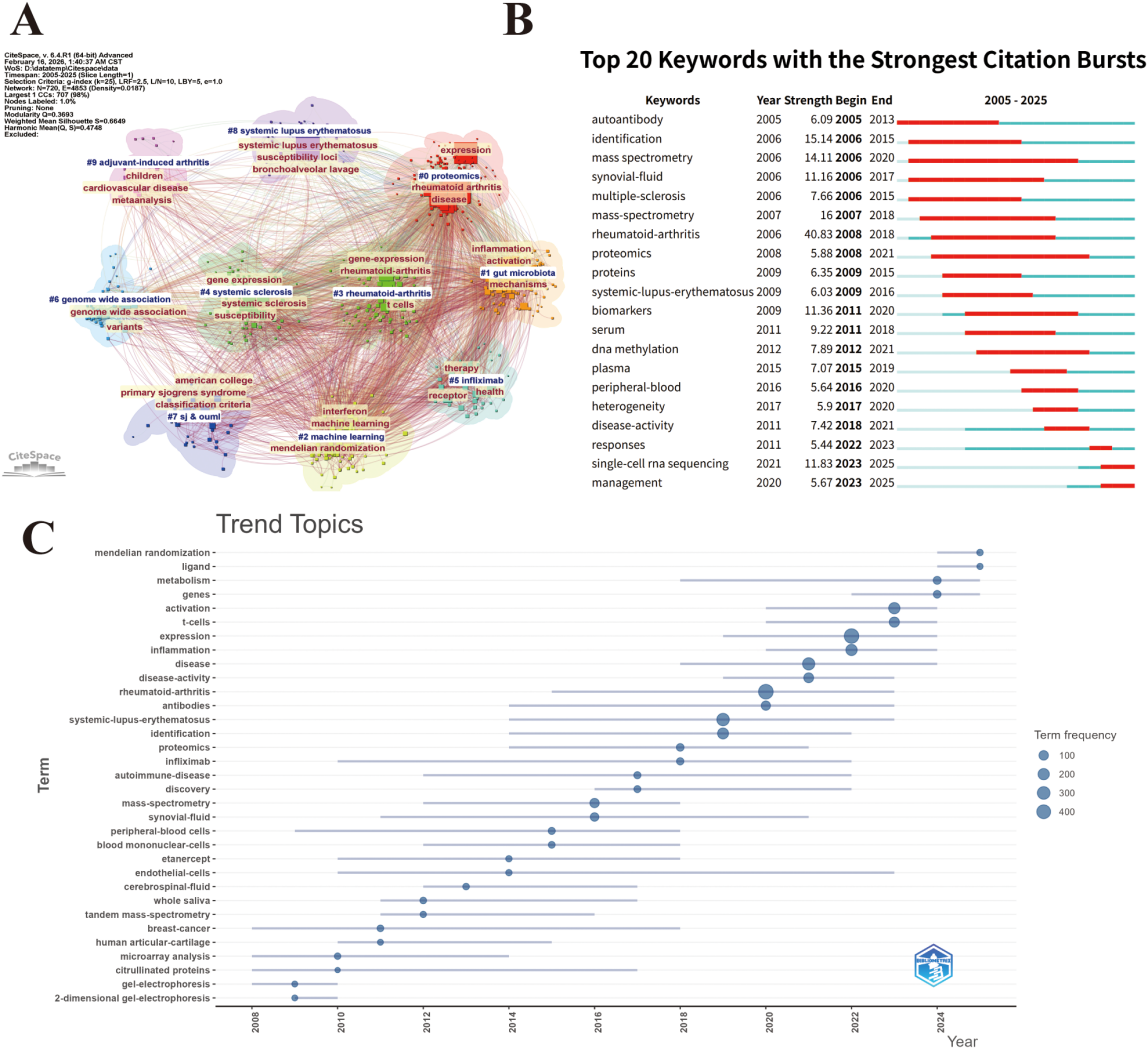
Keyword analysis. **(A)** Clustering network of high-frequency keywords (Nodes represent keywords; node size indicates keyword frequency; links indicate co-occurrence relationships,CiteSpace). **(B)** Top 20 keywords with the strongest citation bursts (Burst detection identifies keywords with rapid increases in usage over time. Red Bars indicate burst duration; “Strength” quantifies burst intensity under CiteSpace burst algorithm). **(C)** Keyword trend analysis over time (Terms represent high-frequency keywords; bubble size indicates frequency within each time slice; placement reflects temporal evolution of topic emphasis.Word Minimum Frequency: 5;Number of Words per Year: 2.R package).

We performed keyword clustering analysis and identified a total of ten clusters, namely #0 proteomics, #1 gut microbiota, #2 machine learning, #3 rheumatoid arthritis, #4 systemic sclerosis, #5 infliximab, #6 genome-wide association, #7 Sj & ouml, #8 systemic lupus erythematosus, and #9 adjuvant-induced arthritis(**Figure 6A**). These clusters collectively illustrate the multidimensional research landscape of the field. Proteomics and genome-wide association studies emphasize the importance of multi-omics approaches in elucidating molecular mechanisms and genetic susceptibility. The emergence of machine learning reflects the increasing integration of artificial intelligence into disease prediction and precision medicine. The gut microbiota represents an expanding frontier, linking environmental factors with immune dysregulation. In addition, rheumatoid arthritis, systemic sclerosis, Sjögren’s syndrome, and systemic lupus erythematosus remain central focuses within SARDs. The presence of infliximab highlights research related to biologic therapies, while adjuvant-induced arthritis reflects the continued reliance on experimental animal models for mechanistic investigations. Overall, the clustering results demonstrate a research framework that integrates molecular profiling, computational analysis, genetic research, therapeutic strategies, and comparative studies of autoimmune diseases.

#### 3.5.3 Keywords burst

Among the burst keywords identified in **Figures 6B**, rheumatoid arthritis exhibited the highest burst strength (Strength = 40.85, 2008–2018), underscoring its position as the central research focus during that period. Subsequently, several high-strength keywords emerged across different stages, reflecting evolving research priorities. Identification (15.14, 2006–2015), mass spectrometry (14.11, 2006–2020), and synovial fluid (11.16, 2006–2017) demonstrated strong and sustained bursts in the early to mid-phase, indicating intensive efforts in molecular detection, protein profiling, and synovial microenvironment analysis. During the subsequent phase, biomarkers (11.36, 2011–2020) showed a prolonged burst, highlighting increasing emphasis on translational applications and diagnostic stratification. More recently, single-cell RNA sequencing (11.83, 2023–2025) has emerged as a prominent burst term, reflecting a methodological shift toward high-resolution cellular heterogeneity analysis and precision medicine approaches. Overall, the burst trend illustrated in **Figure 6B** reveals a clear developmental trajectory: early research concentrated on disease identification and molecular characterization using proteomic technologies, followed by biomarker-driven translational studies, and most recently transitioning toward single-cell technologies and advanced omics methodologies. This evolution indicates a progressive shift from conventional molecular profiling toward high-dimensional, cell-level precision analysis.

#### 3.5.4 Keyword trend

The temporal evolution of research topics in this field from 2008 to 2025 was characterized by distinct stages of development(**Figure 6C**). In the early phase (2008–2012), foundational experimental techniques, including 2-dimensional gel-electrophoresis, gel-electrophoresis, microarray analysis, and citrullinated proteins, dominated the research landscape, reflecting a focus on protein separation and microarray-based detection. From 2013 to 2020, the focus shifted toward molecular mechanisms and clinical translation, with prominent terms such as mass-spectrometry, proteomics, rheumatoid-arthritis, systemic-lupus-erythematosus, antibodies, and disease-activity, alongside therapeutic agents like infliximab and etanercept. After 2021, emerging frontiers emerged, highlighted by mendelian randomization, ligand, metabolism, genes, activation, t-cells, expression, and inflammation, which maintained high term frequencies and extended into 2025. Notably, mass-spectrometry and proteomics exhibited sustained prominence throughout the entire period, underscoring their role as core technical pillars, while the overall trajectory reflected a progressive shift from traditional experimental approaches to multi-omics-driven precision medicine and translational research.

### 3.6 Citation dynamics and paradigm shifts in the field

#### 3.6.1 Citation count analysis

**Figure 7:**
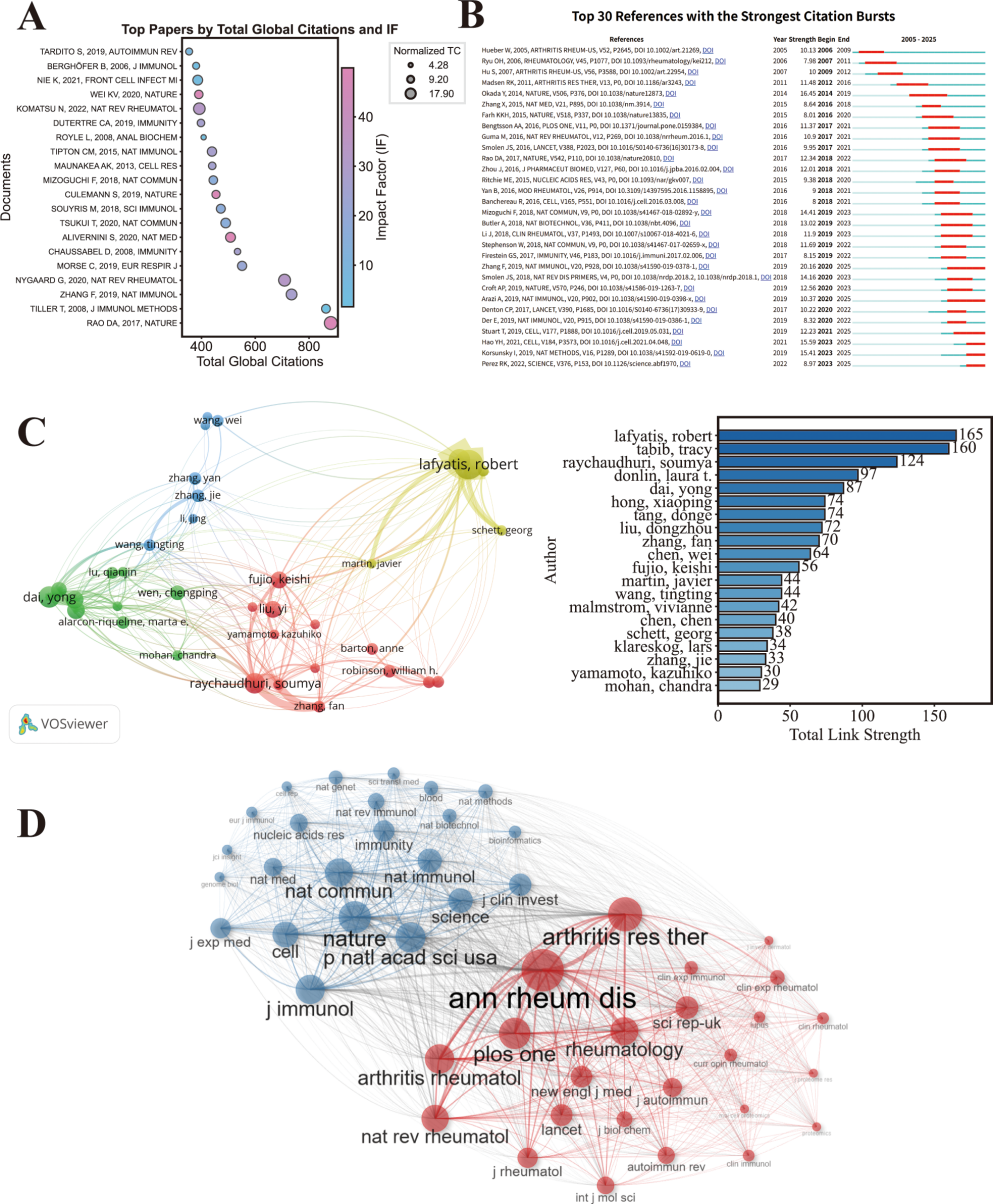
Citation analysis. **(A)** Bubble chart of the top 20 globally cited publications showing citation counts and journal impact factors (Bubble size represents total citations; x-axis represents total Global Citations; y-axis represents papers, Clolor represents journal impact factor,R package). **(B)** Top 30 references with the strongest citation bursts (Red Bars indicate burst duration; “Strength” indicates burst intensity.CiteSpace). **(C)** Author co-citation network with total link strength (Nodes represent cited authors; node size indicates total co-citation counts; links indicate co-citation relationships; link thickness reflects TLS.citation-count threshold ≥ 80.VOSviewer). **(D)** Journal co-citation network (Nodes represent cited journals; node size indicates co-citation frequency; links indicate co-citation relationships,network layout set to Auto; clustering algorithm set to Louvain; limit the number of nodes to 50; repulsion set to 0.5; enable “Remove isolated nodes”; and require that each edge connects at least 2 nodes, R package).

Citation analysis revealed a highly structured and evolving intellectual landscape (**Figure 7**; **Supplementary Table 2**). The top 20 most cited publications were dominated by studies published in high-impact journals, including Nature, NAT IMMUNOL, NAT REV RHEUMATOL et al. The most cited article was Rao DA et al. (14, Nature, 881 citations), followed by fimmu.2026.1759610 Tiller T et al. (15, J Immunol Methods, 863 citations) and Zhang F et al. (17, Nature Immunology, 735 citations). Notably, several recent publications exhibited exceptionally high annual citation rates, such as Nygaard G (16, NAT REV RHEUMATOL, 101.29 citations/year) (**Supplementary Table S2**; **Figure 7A**).

#### 3.6.2 Citation burst

Citation burst analysis further delineated temporal shifts in research emphasis (**Figure 7B**). The top three references by citation burst strength were Defining inflammatory cell states in rheumatoid arthritis joint synovial tissues by integrating single-cell transcriptomics and mass cytometry (17) (Strength 20.16, 2020–2025), Genetics of rheumatoid arthritis contributes to biology and drug discovery (18) (Strength 16.45, 2014–2019), and Integrated analysis of multimodal single-cell data (19) (Strength 15.59, 2023–2025).

#### 3.6.3 Author co-citation network

The author co-citation network (**Figure 7C**) demonstrated a concentrated intellectual core. Lafyatis R. (TLS = 165), Tabib T. (TLS = 160), and Raychaudhuri S. (TLS = 124) occupied central positions within the network, indicating that their work forms a shared conceptual foundation frequently cited together. The dense interconnections among these authors reflect convergence around immune cell heterogeneity and tissue-resolved pathogenesis as dominant research themes.

#### 3.6.4 Journal co-citation network

Journal co-citation analysis (**Figure 7D**) revealed a core aggregation structure of authoritative journals with interdisciplinary integration characteristics. Core cited journals were concentrated in top-tier basic medical science journals (Nature, Immunity, Nature Immunology, Cell) and flagship specialist journals in rheumatology and autoimmunity (Nature Reviews Rheumatology, Arthritis & Rheumatology, Annals of the Rheumatic Diseases), with supplementary distribution in well-regarded interdisciplinary journals (Nucleic Acids Research, Science).

### 3.7 Evolutionary trajectory of research themes

**Figure 8:**
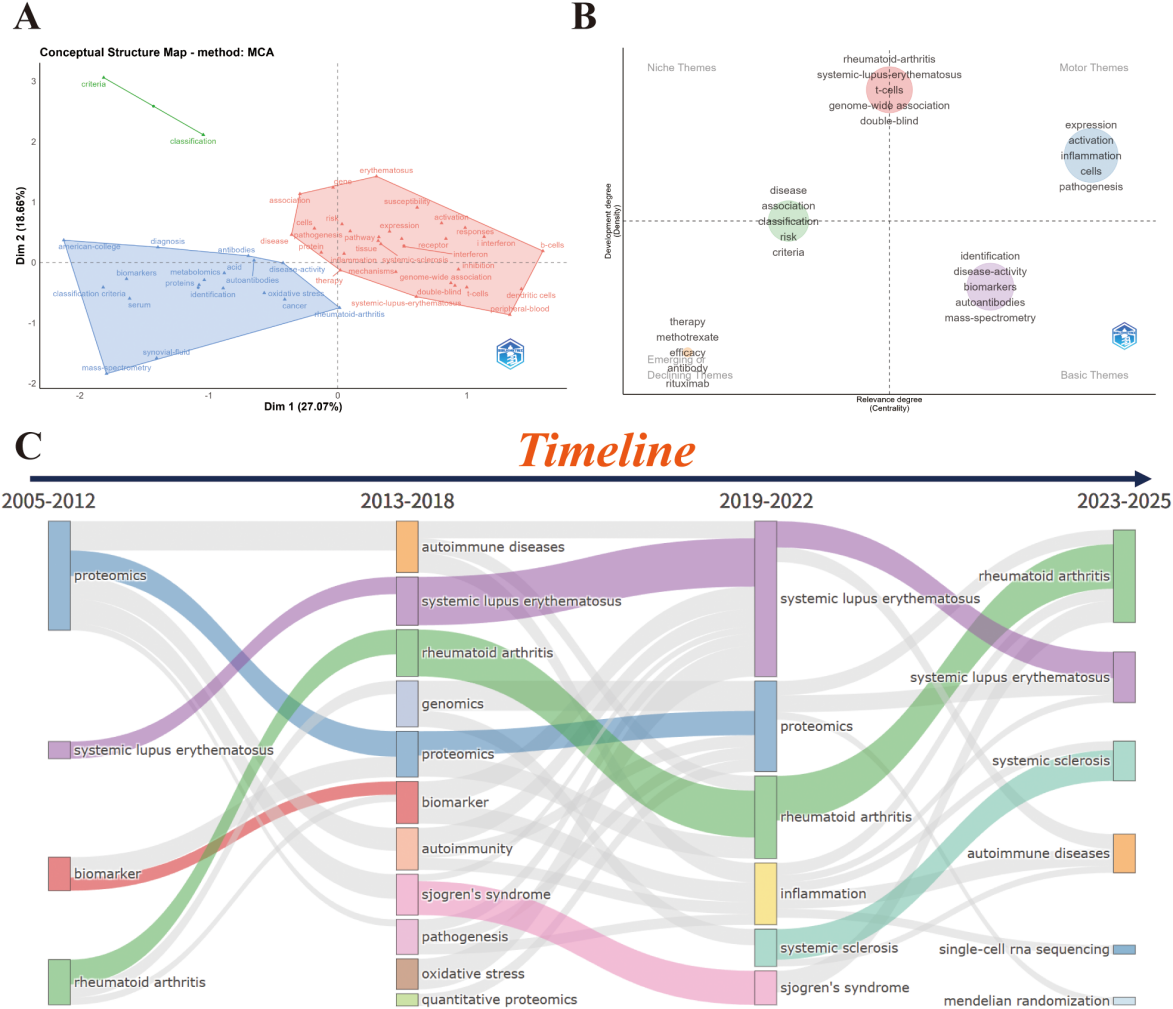
Thematic evolution analysis. **(A)** Multiple Correspondence Analysis (MCA) (Points represent keywords; proximity indicates conceptual similarity based on co-occurrence.R package). **(B)** Thematic map based on centrality and density (Each bubble represents a theme; bubble size indicates number of keywords/publications within the theme. Quadrants correspond to motor themes, basic themes, niche themes, and emerging/declining themes. Extract the top 250 terms; require a minimum frequency of 5 occurrences per 1,000 documents for each cluster; calculate weights using the “inclusion index weighted by term frequency”; and set the minimum weight-index threshold to 0.1.R package). **(C)** Thematic evolution map (Themes are tracked across time slices; links indicate how themes evolve/merge/split between periods. extract the top 250 terms; require a minimum frequency of 5 occurrences per 1,000 documents for each cluster; for visualization, display 3 labels per cluster with label size set to 0.3; construct the network using the Louvain clustering algorithm; and set the community repulsion parameter to 0.5.R package).

To explore the thematic evolution of multi-omics research in SARDs, Multiple Correspondence Analysis (MCA) revealed three distinct thematic groupings: a classification and diagnostic cluster centered on classification criteria, diagnosis, antibodies, biomarkers, and mass spectrometry, reflecting the foundational role of diagnostic and detection methods in the field; a core disease and mechanism cluster encompassing rheumatoid arthritis, systemic lupus erythematosus, disease activity, inflammation, pathogenesis, and therapy, which represents the central focus on disease mechanisms and treatment strategies; and an immunological and genetic susceptibility cluster focusing on b-cells, t-cells, interferon, gene expression, susceptibility, and genome-wide association, highlighting the critical role of immunological and genetic factors in driving disease development and progression. (**Figure 8A**).

Thematic centrality mapping categorized topics into four clusters: Niche Themes (systemic lupus erythematosus, T cells, genome-wide association), Motor Themes (expression, activation, inflammation), Emerging of Declining Themes (therapy、antibody、efficicy,rituximab) and Basic Themes (pathogenesis, biomarkers, therapy, methotrexate) (**Figure 8B**).

The timeline map (**Figure 8C**) exhibits the temporal distribution and evolutionary trends of core research topics in the field across four phases from 2005 to 2025, with the specific objective results as follows:2005–2012: The research topics were primarily concentrated on autoimmune diseases, proteomics、biomarker and rheumatoid arthritis, with systemic lupus erythematosus also emerging as a key research focus in this phase.2013–2018: Systemic lupus erythematosus and rheumatoid arthritis remained the core disease-focused topics; meanwhile, genomics was newly added to the research scope, and proteomics continued to be a prominent technical research theme in this period.2019–2022: The research topics further expanded and refined, with systemic sclerosis, biomarker, autoimmunity and inflammation becoming the key research directions; systemic lupus erythematosus and proteomics still maintained their important research status, and sjogren’s syndrome and pathogenesis also emerged as research hotspots in this phase.2023–2025: The research frontier presented a trend of technical upgrading and in-depth mechanism exploration, with the emergence of single-cell rna sequencing, mendelian randomization and quantitative proteomics as new core topics; rheumatoid arthritis, systemic sclerosis were also included in the key research content of this latest phase.

### 3.8 Validation analysis based on pubmed data

**Figure 9:**
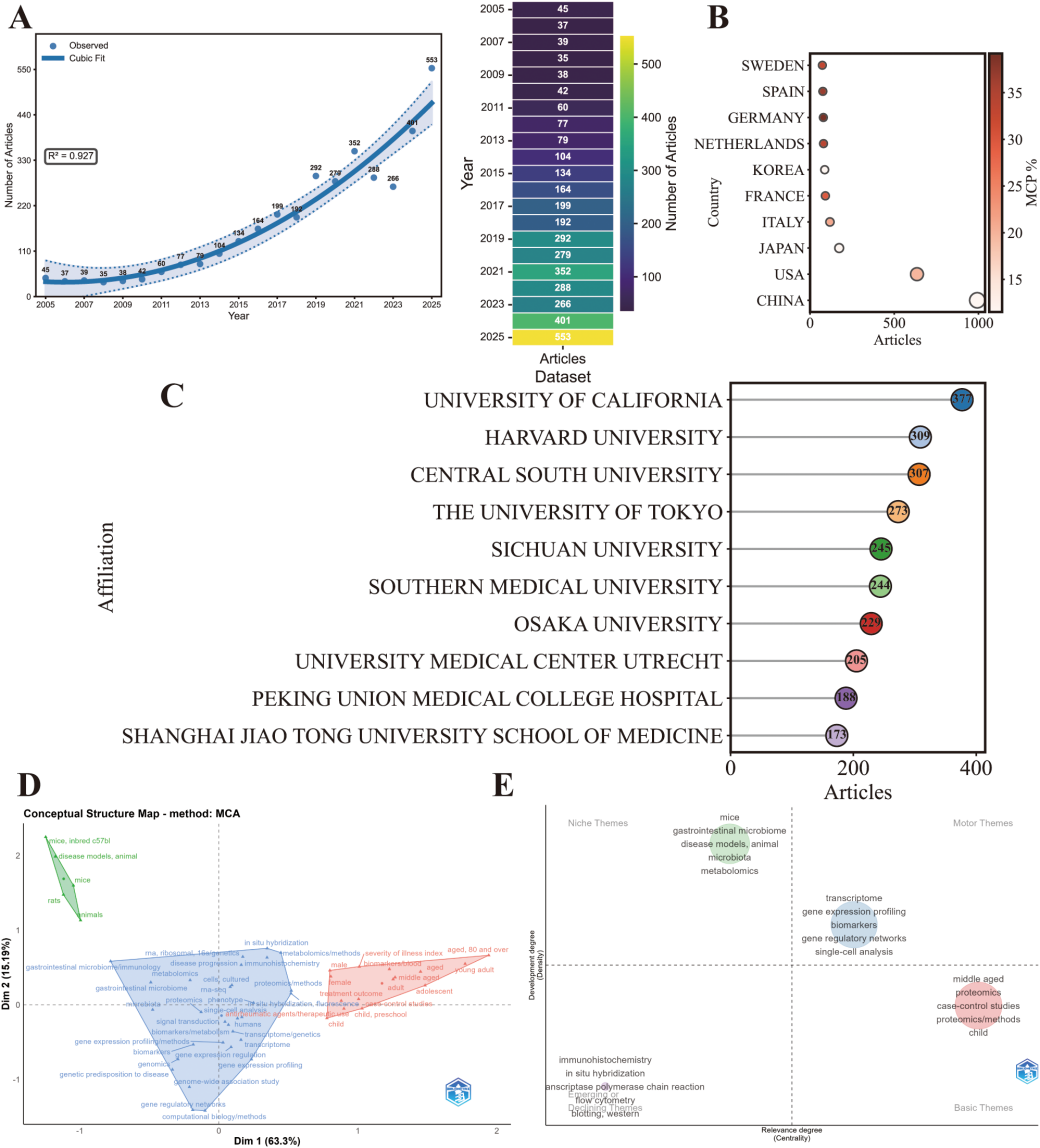
PubMed-based thematic and trend validation. (Based on the PubMed dataset). **(A)** Annual publication trend with fitted curve (R package). **(B)** Bubble chart of the top 10 most productive countries (MCP%) (R package). **(C)** Lollipop chart of the top 10 most productive institutions (R package). **(D)** Multiple Correspondence Analysis (MCA) (R package). **(E)** Thematic map based on centrality and density (extract the top 250 terms; require a minimum frequency of 5 occurrences per 1,000 documents for each cluster; for visualization, display 3 labels per cluster with label size set to 0.3; construct the network using the Louvain clustering algorithm; and set the community repulsion parameter to 0.5,R package).

To validate the robustness of the WoS-based findings, PubMed-indexed studies were analyzed independently (**Figure 9**). The annual publication trend demonstrated sustained growth, with a marked acceleration after 2018 (**Figure 9A**), consistent with the overall expansion observed in the WoS dataset.

At the national level, China ranked first with 993 publications (27%), followed by the United States with 635 publications (17.3%). Japan (173), Italy (118), and France (91) also ranked among the top contributors. In terms of collaboration patterns, Germany (MCP% = 39.2), Spain (36.8), the Netherlands (33.8), and Sweden (32.9) exhibited relatively higher proportions of international co-authorship, whereas China (11.5%) and Korea (11.5%) showed comparatively lower MCP percentages (**Supplementary Table S3**, **Figure 9B**). These findings indicate that European countries demonstrate stronger cross-national collaboration intensity.

Institutional analysis (**Figure 9C**) further confirmed the dominance of leading academic centers, including the University of California(377) and Harvard University(309), alongside major institutions in East Asia. The difference in publication counts of core institutions (e.g., Harvard University) between WoSCC and PubMed datasets is attributed to the different database coverage characteristics and statistical calibers: the WoSCC dataset includes all types of academic publications (research articles, reviews, etc.) that meet the multi-omics definition, while the PubMed dataset is supplemented with clinical study-oriented screening, resulting in partial differences in the included literature.

Multiple Correspondence Analysis (MCA) revealed three distinct thematic groupings (**Figure 9D**): an animal model and microbiome cluster centered on mice, inbred c57bl, disease models, animal, and gastrointestinal microbiome, reflecting the foundational role of animal models and gut microbiota in mechanistic studies; a large, central molecular and omics technology cluster encompassing transcriptome, proteomics, genomics, single-cell analysis, biomarkers, and computational biology/methods, which represents the core methodological and molecular foundation driving the field; and a clinical and population-based research cluster focusing on human subjects, case-control studies, treatment outcomes, and antirheumatic agents, highlighting the strong translational and clinical dimension of the field.

Thematic centrality mapping (**Figure 9E**) categorized topics into four quadrants based on relevance degree (centrality) and development degree (density): niche themes (e.g., mice, gastrointestinal microbiome, metabolomics); motor themes (e.g., transcriptome, biomarkers, single-cell analysis) are highly central and rapidly developing basic themes (e.g., proteomics, case-control studies) are foundational to the field with high centrality; and emerging or declining themes (e.g., immunohistochemistry, reverse transcriptase polymerase chain reaction, blotting, western) represent traditional techniques.

Overall, the PubMed validation analysis confirms the sustained growth trend, geographic concentration in China and the United States, stronger international collaboration among European countries, and the central role of transcriptomics and single-cell technologies. The convergence of results across databases strengthens the reliability of the identified research patterns.

Using the PubMed filter “Randomized Controlled Trial” and applying the predefined integrative multi-omics criteria, 8 eligible RCT-related publications were identified (**Supplementary Table 4**). These studies were published between 2018 and 2025, and mainly involved rheumatoid arthritis (RA, n = 3) and early RA (n = 2), with additional trials in systemic lupus erythematosus (SLE, n = 2) and Sjögren’s syndrome (n = 1). In terms of multi-omics modalities, the RCT subset commonly combined gene expression/transcriptomic profiling with proteomic readouts and proteomic or metabolomic biomarker profiling to characterize treatment response and remission trajectories. Overall, the limited number and heterogeneous designs of eligible RCT-related studies suggest that interventional validation of integrative multi-omics in SARDs remains relatively early compared with the rapidly expanding observational literature.

## 4 Discussion

### 4.1 Research pattern of multi-omics in the field of SARDs

Over the past years, multi-omics technology has experienced rapid development in the field of SARDs research, showing a significant upward trend (20)). The academic influence of multi-omics research in the field of SARDs has been continuously expanding, covering multiple disciplines such as immunology, genetics, biochemistry, and bioinformatics (21–25). Our results show a sustained and accelerating expansion of multi-omics research in SARDs from 2005 to 2025, with annual publications increasing from 11 to 525 and a marked growth surge after 2021. This pattern supports a clear transition of the field from an early exploratory stage to rapid expansion, indicating increasing consolidation of multi-omics as a mainstream research strategy in SARDs.

Based on country–institution networks and collaboration indicators, the global research pattern is dominated by China, the United States, and several European countries, with Europe showing comparatively higher international collaboration intensity (MCP% up to 39.2%). The extensive distribution of national and institutional cooperation networks, especially the formation of three major cooperation circles (North America-Europe-Asia Pacific), provides organizational guarantee for the cross-regional and interdisciplinary integration of multi-omics technologies. Despite overall growth, substantial regional disparities were observed in publication output and collaboration intensity. The United States, China, and developed European countries occupy a dominant position in terms of publication output, citation frequency, and international cooperation. China ranks first in terms of publication output and has become an important research force in this field. However, compared with the United States, there is still a gap in terms of TLS with other countries, and there is room for improvement in international academic influence.

The dual-map overlay shows dominant citation pathways from Medicine/Clinical journals to Molecular Biology/Genetics, indicating that SARD-related clinical questions increasingly draw upon mechanistic and multi-omics evidence. This structural linkage helps contextualize the observed shift toward single-cell and integrative omics themes in recent years.

### 4.2 Application value and differential contributions of multi-omics technologies in SARDs

Our keyword frequency and clustering results indicate that multi-omics applications in SARDs are primarily concentrated in representative diseases (e.g., RA, SLE, SS), with major emphases on molecular stratification, biomarker discovery, and immune-pathway characterization.

In RA research, for example, the application of multi-omics technology presents multi-dimensional collaborative characteristics: Genomics has identified the association between key genes such as HLA-DRB1 and PTPN22 and genetic susceptibility to the disease through genome-wide association studies (GWAS), providing molecular clues for tracing the origin of pathogenesis (26). Single-cell transcriptomics has enabled the accurate localization and functional annotation of core inflammatory cell subsets such as pro-inflammatory fibroblasts and macrophages in synovial tissue (27), which has greatly deepened the understanding of the mechanism underlying the formation of the local inflammatory microenvironment in joints. Proteomics-protein interaction network analysis has identified three core genes of RA, namely fibronectin 1 (FN1), acetylcholinesterase (ACHE), and aquaporin 1 (AQP1), as biological response markers (28). Moreover, the integrated analysis of multi-omics data has overcome the limitations of single technologies and shown significant advantages in the long-term prognostic assessment of joint erosion risk and the accurate prediction of the efficacy of biological agents (29).

For SLE, multi-omics technology has also promoted the depth and precision of research: Transcriptomics studies have confirmed that the abnormal continuous activation of the type I interferon pathway is a core molecular feature throughout the disease process and is closely related to the occurrence of immune disorders (30). Metabolomics has revealed significant disorders in the tryptophan metabolism pathway. The levels of metabolites such as kynurenine and 3-hydroxykynurenine in the serum of patients are increased, and the abnormal kynurenine/tryptophan ratio can be used as a potential disease monitoring marker. Its change mechanism is related to cellular oxidative stress and immune cell dysfunction (31). Genomics has laid the foundation for individualized diagnosis and treatment by analyzing the genetic correlation of genes such as AHNAK2, CSK, IKBKB, IRAK1, NCF2, OAS1, TYK2, and WDFY4(X. 32). Single-cell RNA sequencing (scRNA-seq) has revealed SLE cell type-specific molecules and genetic associations (33), cellular heterogeneity of skin lesions (34), and disease diagnosis and prognostic markers (35) at the single-cell level.

### 4.3 Evolution logic of research hotspots

The temporal evolution of keywords and literature clusters clearly presents the internal logic of research hotspots in the field. In the early stage (2005-2010), the basic application of omics technologies such as proteomics and metabolomics was the core, focusing on the molecular characteristic analysis of samples such as synovial fluid and serum (36). This stage was the “technology foundation period” of multi-omics in this field, accumulating methodological and data foundations for subsequent research. In the middle stage (2010-2020), the research shifted to the exploration of epigenetics (DNA methylation) (37), microecology (gut microbiota) (38), and metabolic mechanisms (39–41). The research perspective expanded from the “single molecular level” to the “multi-molecular network level”, and began to touch the complex regulatory mechanisms of SARDs In the recent stage (since 2021), single-cell RNA sequencing technology (42–44) has become the core, promoting research to advance to “cell heterogeneity and functional differentiation at the single-cell resolution”. This stage is the “precision breakthrough period” of the field, providing a key tool for revealing the dynamic role of different cell subsets in the disease microenvironment.

### 4.4 Existing challenges and future breakthroughs

Although multi-omics has expanded rapidly in SARD research, several barriers continue to constrain integration and translation. At the data level, the standardization and integration of multi-omics data is still a core problem—there are significant differences in experimental procedures, data formats, and analysis methods between different omics technologies, making it difficult to efficiently integrate multi-omics data and unable to give full play to the advantages of “systematic analysis” (45–47). At the sample level, most studies have a limited sample size and lack the support of multi-center, long-term follow-up cohorts, which affects the universality of biomarkers and the reliability of prognosis models (46, 48). At the clinical translation level, a large number of potential biomarkers and therapeutic targets discovered by multi-omics are difficult to truly enter clinical practice due to the lack of standardized verification procedures and clinical evaluation systems (46, 47, 49).

Importantly, the PubMed-derived RCT subset remains small (n = 8) and is concentrated in RA/early RA, with fewer trials in SLE and Sjögren’s syndrome. This imbalance implies that most multi-omics work in SARDs continues to be discovery-oriented and mechanistic, whereas rigorous interventional validation and biomarker-guided trial designs are still emerging. Future translational progress may benefit from prospectively designed, multi-center trials that predefine integrative pipelines, harmonize omics platforms, and link multi-omics–derived stratification to clinically actionable endpoints, thereby enabling clearer evaluation of clinical utility (50, 51).

### 4.5 Known bibliometric biases and interpretive constraints

Bibliometric analyses are subject to several well-recognized structural biases. First, citation age bias may favor older publications that have had more time to accumulate citations, potentially underrepresenting the impact of recent high-quality studies. Second, self-citation practices and collaborative citation networks may artificially inflate citation counts for certain authors or institutions (52, 53). Third, differences in field size and publication culture across disciplines can influence citation density, potentially affecting cross-topic comparisons (54). Fourth, English-language and high-impact journal indexing biases inherent to WoSCC may limit representation of regional or non-English scholarship. Although normalization procedures and multi-tool validation were implemented to mitigate these effects, such biases are intrinsic to bibliometric methodology and should be considered when interpreting relative influence and trend dynamics (55).

### 4.6 Methodological strengths and limitations

Compared with conventional narrative or disease-specific reviews that primarily summarize mechanistic findings or clinical evidence, the present bibliometric approach provides a quantitative macro-structural perspective on the field. By analyzing publication volume, collaboration networks, and thematic evolution across 2,576 records, this study identifies developmental phases, collaboration intensity patterns, and shifts toward single-cell–driven immune heterogeneity research that may be less readily captured through traditional review methodologies.

This study improves construct validity by operationally defining multi-omics as the integration of at least two omics layers and implementing independent manual validation (Cohen’s kappa = 0.92). Systematic data cleaning, disambiguation procedures, and standardized script-based workflows enhanced methodological transparency and reproducibility. The complementary PubMed validation further strengthened the robustness of thematic findings.

However, several limitations should be acknowledged. First, reliance primarily on WoSCC may introduce database coverage bias. Although WoSCC is authoritative and widely used in bibliometric research, it does not index all journals globally, and non-English publications, regional journals, and conference proceedings may be underrepresented. Second, although multi-omics was operationally defined as integration of at least two omics layers within a single analytical framework, terminology heterogeneity in the literature may still introduce residual misclassification. A random validation sample indicated that approximately 15% of retrieved studies did not fully meet the integrative definition, suggesting a modest degree of over-retrieval bias. Third, bibliometric analysis identifies structural associations, co-occurrence relationships, and temporal trajectories but does not establish causal relationships between technological developments, research themes, or clinical translation. Interpretations linking technological breakthroughs to downstream translational impact should therefore be regarded as hypothesis-generating rather than confirmatory. Fourth, citation-based indicators are subject to time-lag effects and may disadvantage recently published studies. Finally, the rapid evolution of omics technologies means that emerging concepts may not yet be fully reflected in database indexing systems.

These methodological considerations should be taken into account when interpreting the scope, representativeness, and temporal dynamics of the findings.

## 5 Conclusion

Multi-omics research in SARDs has evolved from bulk molecular profiling toward high-resolution immune cell–level investigation, with rapid publication growth after 2021 and increasing thematic concentration on immune heterogeneity and biomarker stratification. By mapping developmental phases, collaboration structures, and thematic evolution, this study provides a quantitative macro-level overview of the field’s current maturity and emerging directions.

This work offers a bibliometric synthesis that complements conventional reviews by quantifying publication dynamics, identifying influential contributors and journals, and visualizing thematic transitions. The resulting maps and indicators may help researchers contextualize mechanistic findings within broader structural and temporal trends. Methodologically, the bibliometric approach provides advantages in large-scale quantitative mapping, reproducible workflow implementation, and visualization of collaboration networks and thematic evolution across thousands of publications. However, it also has inherent disadvantages: it depends on database coverage and indexing policies, is influenced by citation-related biases (e.g., citation-age effects and self-citation), and cannot establish causal relationships or directly evaluate mechanistic validity or clinical efficacy. Therefore, the findings should be interpreted as macro-structural and developmental insights that complement, rather than replace, evidence synthesis and experimental validation.

Future progress will likely depend on improved standardization of integrative pipelines, validation in larger multi-center cohorts, and clearer implementation pathways to translate multi-omics discoveries into clinically actionable tools.

## Data Availability

The original contributions presented in the study are included in the article/**Supplementary Material**, further inquiries can be directed to the corresponding author/s.

## Supplementary material

Supplementary Table 1 Search strategies for WoSCC and PubMed.

Supplementary Table 2 Top 20 globally cited publications and detailed information.

Supplementary Table 3 Top 10 most productive countries in PubMed and their collaboration rates.

Supplementary Table 4 Analysis of selected randomized controlled trials (RCTs) identified from PubMed.

## References

[1] Berghen N Vulsteke JB Westhovens R Lenaerts J De Langhe E . Rituximab in systemic autoimmune rheumatic diseases: Indications and practical use. Acta Clin Belg. (2019) 74:272–9. doi: 10.1080/17843286.2018.1521904. PMID: 30253707

[2] Nakken B Alex P Munthe L Szekanecz Z Szodoray P . Immune-regulatory mechanisms in systemic autoimmune and rheumatic diseases. Clin Dev Immunol. (2012) 2012:957151. doi: 10.1155/2012/957151. PMID: 22474487 PMC3310204

[3] Ruscitti P Allanore Y Baldini C Barilaro G Bartoloni Bocci E Bearzi P . Tailoring the treatment of inflammatory rheumatic diseases by a better stratification and characterization of the clinical patient heterogeneity. Findings from a systematic literature review and experts' consensus. Autoimmun Rev. (2024) 23:103581. doi: 10.1016/j.autrev.2024.103581. PMID: 39069240

[4] Hayes CN Nakahara H Ono A Tsuge M Oka S . From omics to multi-omics: A review of advantages and tradeoffs. Genes (Basel). (2024) 15:1551. doi: 10.3390/genes15121551. PMID: 39766818 PMC11675490

[5] Ninkov A Frank JR Maggio LA . Bibliometrics: Methods for studying academic publishing. Perspect Med Educ. (2022) 11:173–6. doi: 10.1007/s40037-021-00695-4. PMID: 34914027 PMC9240160

[6] Lazarides MK Lazaridou IZ Papanas N . Bibliometric analysis: Bridging informatics with science. Int J Low Extrem Wounds. (2025) 24:515–7. doi: 10.1177/15347346231153538. PMID: 36710511

[7] Falagas ME Pitsouni EI Malietzis GA Pappas G . Comparison of pubMed, scopus, web of science, and google scholar: strengths and weaknesses. FASEB J. (2008) 22:338–42. doi: 10.1096/fj.07-9492LSF. PMID: 17884971

[8] Pitre T Kirsh S Jassal T Anderson M Padoan A Xiang A . The impact of blinding on trial results: A systematic review and meta-analysis. Cochrane Evid Synth Methods. (2023) 1:e12015. doi: 10.1002/cesm.12015. PMID: 40475370 PMC11795910

[9] Robinson KA Dickersin K . Development of a highly sensitive search strategy for the retrieval of reports of controlled trials using PubMed. Int J Epidemiol. (2002) 31:150–3. doi: 10.1093/ije/31.1.150. PMID: 11914311

[10] Yang H Lee HJ . Research trend visualization by MeSH terms from PubMed. Int J Environ Res Public Health. (2018) 15:1113. doi: 10.3390/ijerph15061113. PMID: 29848974 PMC6025283

[11] Liu B Zhou CJ Ma HW Gong B . Mapping the youth soccer: A bibliometrix analysis using R-tool. Digit Health. (2023) 9:20552076231183550. doi: 10.1177/20552076231183550. PMID: 37361439 PMC10286214

[12] Arruda H Silva ER Lessa M Proença D Bartholo R . VOSviewer and bibliometrix. J Med Libr Assoc. (2022) 110:392–5. doi: 10.5195/jmla.2022.1434. PMID: 36589296 PMC9782747

[13] Synnestvedt MB Chen C Holmes JH . CiteSpace II: Visualization and knowledge discovery in bibliographic databases. AMIA Annu Symp Proc. (2005) 2005:724–8. PMC1560567 16779135

[14] Rao DA Gurish MF Marshall JL Slowikowski K Fonseka CY Liu Y . Pathologically expanded peripheral T helper cell subset drives B cells in rheumatoid arthritis. Nature. (2017) 542(7639):110-14. doi: 10.1038/nature20810. PMID: 28150777 PMC5349321

[15] Tiller T Meffre E Yurasov S Tsuiji M Nussenzweig MC Wardemann H. . Efficient generation of monoclonal antibodies from single human B cells by single cell RT-PCR and expression vector cloning. J Immunol Methods. (2008) 329 (1-2):112–124. doi: 10.1016/j.jim.2007.09.017. PMID: 17996249 PMC2243222

[16] Nygaard G Firestein GS . Restoring synovial homeostasis in rheumatoid arthritis by targeting fibroblast-like synoviocytes. Nat Rev Rheumatol. (2020) 16:316–33. doi: 10.1038/s41584-020-0413-5. PMID: 32393826 PMC7987137

[17] Zhang F Wei K Slowikowski K Fonseka CY Rao DA Kelly S . Defining inflammatory cell states in rheumatoid arthritis joint synovial tissues by integrating single-cell transcriptomics and mass cytometry. Nat Immunol. (2019) 20:928–42. doi: 10.1038/s41590-019-0378-1. PMID: 31061532 PMC6602051

[18] Okada Y Wu D Trynka G Raj T Terao C Ikari K . Genetics of rheumatoid arthritis contributes to biology and drug discovery. Nature. (2014) 506:376–81. doi: 10.1038/nature12873. PMID: 24390342 PMC3944098

[19] Hao Y Hao S Andersen-Nissen E Mauck WM Zheng S Butler A . Integrated analysis of multimodal single-cell data. Cell. (2021) 184:3573–3587.e3529. doi: 10.1016/j.cell.2021.04.048. PMID: 34062119 PMC8238499

[20] Subramanian I Verma S Kumar S Jere A Anamika K . Multi-omics data integration, interpretation, and its application. Bioinform Biol Insights. (2020) 14:1177932219899051. doi: 10.1177/1177932219899051. PMID: 32076369 PMC7003173

[21] Huang L Shi J Li H Lin Q . Integrated multi-omics analysis reveals diagnostic biomarkers and therapeutic targets for systemic lupus erythematosus. Med (Baltimore). (2025) 104:e42290. doi: 10.1097/md.0000000000042290. PMID: 40826785 PMC12367050

[22] Jian C Zhu J Wu J Zhang Y Chen J Wang H . Multiomics analyses reveal an essential role of tryptophan in treatment of csDMARDs in rheumatoid arthritis. Adv Sci (Weinh). (2025) 13:e13170. doi: 10.1002/advs.202413170. PMID: 40985320 PMC12904049

[23] Liang L Liang H He M Zhang H Ke P . Integrative multi-omics analysis reveals the interaction mechanisms between gut microbiota metabolites and ferroptosis in rheumatoid arthritis. Front Immunol. (2025) 16:1608262. doi: 10.3389/fimmu.2025.1608262. PMID: 40703513 PMC12283288

[24] Xiao Y Xie S Liu Y Jiang Y Li H Zhang H . Multiomics analysis uncovers subtype-specific mechanisms and biomarkers in idiopathic inflammatory myopathies. Ann Rheum Dis. (2025) 85:172–85. doi: 10.1016/j.ard.2025.08.011. PMID: 40935702

[25] Yin Z Luo S Qin Z Song J Zhang L Zhang G . Integrative multi-omics reveals that Salvia miltiorrhiza active fraction ameliorates rheumatoid arthritis in rats via inhibiting inflammation and ferroptosis. Phytomedicine. (2025) 148:157258. doi: 10.1016/j.phymed.2025.157258. PMID: 40975040

[26] Kurkó J Besenyei T Laki J Glant TT Mikecz K Szekanecz Z . Genetics of rheumatoid arthritis - a comprehensive review. Clin Rev Allergy Immunol. (2013) 45:170–9. doi: 10.1007/s12016-012-8346-7. PMID: 23288628 PMC3655138

[27] Cheng L Wang Y Wu R Ding T Xue H Gao C . New insights from single-cell sequencing data: Synovial fibroblasts and synovial macrophages in rheumatoid arthritis. Front Immunol. (2021) 12:709178. doi: 10.3389/fimmu.2021.709178. PMID: 34349767 PMC8326910

[28] Jian C Wei L Wu T Li S Wang T Chen J . Comprehensive multi-omics analysis reveals the core role of glycerophospholipid metabolism in rheumatoid arthritis development. Arthritis Res Ther. (2023) 25:246. doi: 10.1186/s13075-023-03208-2. PMID: 38102690 PMC10722724

[29] Tasaki S Suzuki K Kassai Y Takeshita M Murota A Kondo Y . Multi-omics monitoring of drug response in rheumatoid arthritis in pursuit of molecular remission. Nat Commun. (2018) 9:2755. doi: 10.1038/s41467-018-05044-4. PMID: 30013029 PMC6048065

[30] Baker T Sharifian H Newcombe PJ Gavin PG Lazarus MN Ramaswamy M . Type I interferon blockade with anifrolumab in patients with systemic lupus erythematosus modulates key immunopathological pathways in a gene expression and proteomic analysis of two phase 3 trials. Ann Rheum Dis. (2024) 83:1018–27. doi: 10.1136/ard-2023-225445. PMID: 38569851 PMC12056589

[31] Liu Y Yang X . A review on the novel biomarkers of systemic lupus erythematosus discovered via metabolomic profiling. Front Immunol. (2024) 15:1443440. doi: 10.3389/fimmu.2024.1443440. PMID: 39569194 PMC11576423

[32] Yin X Kim K Suetsugu H Bang SY Wen L Koido M . Meta-analysis of 208370 East Asians identifies 113 susceptibility loci for systemic lupus erythematosus. Ann Rheum Dis. (2021) 80:632–40. doi: 10.1136/annrheumdis-2020-219209. PMID: 33272962 PMC8053352

[33] Perez RK Gordon MG Subramaniam M Kim MC Hartoularos GC Targ S . Single-cell RNA-seq reveals cell type-specific molecular and genetic associations to lupus. Science. (2022) 376:eabf1970. doi: 10.1126/science.abf1970. PMID: 35389781 PMC9297655

[34] Zheng M Hu Z Mei X Ouyang L Song Y Zhou W . Single-cell sequencing shows cellular heterogeneity of cutaneous lesions in lupus erythematosus. Nat Commun. (2022) 13:7489. doi: 10.1038/s41467-022-35209-1. PMID: 36470882 PMC9722937

[35] Li Y Ma C Liao S Qi S Meng S Cai W . Combined proteomics and single cell RNA-sequencing analysis to identify biomarkers of disease diagnosis and disease exacerbation for systemic lupus erythematosus. Front Immunol. (2022) 13:969509. doi: 10.3389/fimmu.2022.969509. PMID: 36524113 PMC9746895

[36] Al Kindi MA Colella AD Chataway TK Jackson MW Wang JJ Gordon TP . Secreted autoantibody repertoires in Sjögren's syndrome and systemic lupus erythematosus: A proteomic approach. Autoimmun Rev. (2016) 15:405–10. doi: 10.1016/j.autrev.2016.01.008. PMID: 26804757

[37] Zhu H Wu LF Mo XB Lu X Tang H Zhu XW . Rheumatoid arthritis-associated DNA methylation sites in peripheral blood mononuclear cells. Ann Rheum Dis. (2019) 78:36–42. doi: 10.1136/annrheumdis-2018-213970. PMID: 30297333

[38] Zhu JH Wu LP Deng L Zang SG Li XB Chen X . Gut microbiota and metabolism in systemic lupus erythematosus: from dysbiosis to targeted interventions. Eur J Med Res. (2025) 30:971. doi: 10.1186/s40001-025-03264-1. PMID: 41088420 PMC12522637

[39] Zhu C Xu J He S Niu Q Liu Y Guo X . A longitudinal study reveals metabolomic markers for individuals at risk, disease severity, and treatment response in rheumatoid arthritis. Adv Sci (Weinh). (2025) 12:e04414. doi: 10.1002/advs.202504414. PMID: 40801465 PMC12520550

[40] Anderson JR Chokesuwattanaskul S Phelan MM Welting TJM Lian LY Peffers MJ . (1)H NMR metabolomics identifies underlying inflammatory pathology in osteoarthritis and rheumatoid arthritis synovial joints. J Proteome Res. (2018) 17:3780–90. doi: 10.1021/acs.jproteome.8b00455. PMID: 30229649 PMC6220363

[41] Srivastava NK Sharma S Sharma R Sinha N Mandal SK Sharma D . Metabolic fingerprinting of joint tissue of collagen-induced arthritis (CIA) rat: *In vitro*, high resolution NMR (nuclear magnetic resonance) spectroscopy based analysis. Excli J. (2018) 17:257–72. doi: 10.17179/excli2017-938. PMID: 29743863 PMC5938536

[42] Guo C Liu Q Zong D Zhang W Zuo Z Yu Q . Single-cell transcriptome profiling and chromatin accessibility reveal an exhausted regulatory CD4+ T cell subset in systemic lupus erythematosus. Cell Rep. (2022) 41:111606. doi: 10.1016/j.celrep.2022.111606. PMID: 36351407

[43] Lin SY Yu Y Nie D Yang L Chen Y Chen Y . Single-cell RNA sequencing reveals the heterogeneity and regulatory modules of cell-specific RNA-binding proteins in patients with systemic lupus erythematosus. Biochem Biophys Rep. (2025) 42:101977. doi: 10.1016/j.bbrep.2025.101977. PMID: 40212811 PMC11985018

[44] Zhu H Xiao Y Xie S Meng Q Ding T Huang T . Characterization of muscle tissue cell diversity and clinical implications in idiopathic inflammatory myopathy. J Cachexia Sarcopenia Muscle. (2025) 16:e70043. doi: 10.1002/jcsm.70043. PMID: 40874258 PMC12391830

[45] Airoldi M Remori V Fasano M . Statistical methods for multi-omics analysis in neurodevelopmental disorders: From high dimensionality to mechanistic insight. Biomolecules. (2025) 15:1401. doi: 10.3390/biom15101401. PMID: 41154630 PMC12562612

[46] Higdon R Earl RK Stanberry L Hudac CM Montague E Stewart E . The promise of multi-omics and clinical data integration to identify and target personalized healthcare approaches in autism spectrum disorders. Omics. (2015) 19:197–208. doi: 10.1089/omi.2015.0020. PMID: 25831060 PMC4389910

[47] Wekesa JS Kimwele M . A review of multi-omics data integration through deep learning approaches for disease diagnosis, prognosis, and treatment. Front Genet. (2023) 14:1199087. doi: 10.3389/fgene.2023.1199087. PMID: 37547471 PMC10398577

[48] Dagley LF Emili A Purcell AW . Application of quantitative proteomics technologies to the biomarker discovery pipeline for multiple sclerosis. Proteomics Clin Appl. (2013) 7:91–108. doi: 10.1002/prca.201200104. PMID: 23112123

[49] Lee Y Lee M Shin Y Kim K Kim T . Spatial omics in clinical research: A comprehensive review of technologies and guidelines for applications. Int J Mol Sci. (2025) 26:3949. doi: 10.3390/ijms26093949. PMID: 40362187 PMC12071594

[50] Vainchtein ID Alsema AM Dubbelaar ML Grit C Vinet J van Weering HRJ . Characterizing microglial gene expression in a model of secondary progressive multiple sclerosis. Glia. (2023) 71:588–601. doi: 10.1002/glia.24297. PMID: 36377669 PMC10100411

[51] Yang B Yang Y Wang M Su X . MRGCN: Cancer subtyping with multi-reconstruction graph convolutional network using full and partial multi-omics dataset. Bioinformatics. (2023) 39:btad353. doi: 10.1093/bioinformatics/btad353. PMID: 37255323 PMC10279523

[52] Masic I Jankovic SM . Inflated co-authorship introduces bias to current scientometric indices. Med Arch. (2021) 75:248–55. doi: 10.5455/medarh.2021.75.248-255. PMID: 34759443 PMC8563053

[53] Urlings MJE Duyx B Swaen GMH Bouter LM Zeegers MP . Citation bias and other determinants of citation in biomedical research: Findings from six citation networks. J Clin Epidemiol. (2021) 132:71–8. doi: 10.1016/j.jclinepi.2020.11.019. PMID: 33278612

[54] Seglen PO . Use of citation analysis and other bibliometric methods in evaluation of the quality of research. Tidsskr Nor Laegeforen. (1989) 109:3229–4. 2595690

[55] Szomszor M Adams J Fry R Gebert C Pendlebury DA Potter RWK . Interpreting bibliometric data. Front Res Metr Anal. (2020) 5:628703. doi: 10.3389/frma.2020.628703. PMID: 33870066 PMC8025976

