# Supplementary material for: Application of multi-omics in systemic autoimmune rheumatic diseases: a bibliometric and visualization analysis
Source: Front Immunol. 2026 Apr 16;17:1759610. doi: 10.3389/fimmu.2026.1759610 (PMC13128600; doi:10.3389/fimmu.2026.1759610)
Supplement: Supplementary Table 1 — Search strategies for WoSCC and PubMed. [file SupplementaryFile1.docx]

**Table S1.** Retrieval strategy of publications in the study

| Citation index | |
| --- | --- |
| Web of Science Core Collection (WoSCC) | #TS1= ("autoimmune rheumatic disease*" OR "connective tissue disease*") OR ("rheumatoid arthritis" OR "systemic lupus erythematosus" OR "Sjogren's syndrome" OR "systemic sclerosis" OR "scleroderma") OR ("inflammatory myopathy" OR "polymyositis" OR "dermatomyositis" OR "antisynthetase syndrome") OR("systemic vasculitis" OR "ANCA-associated vasculitis" OR "granulomatosis with polyangiitis" OR "microscopic polyangiitis" OR "eosinophilic granulomatosis with polyangiitis") OR ("antiphospholipid syndrome" OR "mixed connective tissue disease" OR "undifferentiated connective tissue disease")  #TS2= ("multi-omics" OR multiomics OR "integrated omics" OR "omics integration") OR (genomics OR transcriptomics OR proteomics OR metabolomics OR epigenomics OR lipidomics OR glycomics OR metagenomics OR microbiomics) OR ("single cell" OR "single-cell" OR "scRNA-seq" OR "single cell RNA sequencing" OR "single nucleus RNA sequencing" OR "snRNA-seq") OR ("spatial transcriptom*" OR "spatial omics")  TS=TS1 AND TS2 |
| Pubmed | ( "Arthritis, Rheumatoid"[Mesh] OR "Lupus Erythematosus, Systemic"[Mesh] OR "Sjögren's Syndrome"[Mesh] OR "Scleroderma, Systemic"[Mesh] OR "Myositis"[Mesh] OR "Polymyositis"[Mesh] OR "Dermatomyositis"[Mesh] OR "Antisynthetase Syndrome"[Supplementary Concept] OR "Vasculitis"[Mesh] OR "Antineutrophil Cytoplasmic Antibody-Associated Vasculitis"[Mesh] OR "Granulomatosis with Polyangiitis"[Mesh] OR "Microscopic Polyangiitis"[Mesh] OR "Eosinophilic Granulomatosis with Polyangiitis"[Mesh] OR "Antiphospholipid Syndrome"[Mesh] OR "Mixed Connective Tissue Disease"[Mesh] OR "Connective Tissue Diseases"[Mesh] ) AND ( "Genomics"[Mesh] OR "Transcriptome"[Mesh] OR "Proteomics"[Mesh] OR "Metabolomics"[Mesh] OR "Epigenomics"[Mesh] OR "Lipidomics"[Mesh] OR "Glycomics"[Mesh] OR "Metagenomics"[Mesh] OR "Microbiota"[Mesh] OR "Single-Cell Analysis"[Mesh] OR "RNA-Seq"[Mesh] OR "Spatial Transcriptomics"[Mesh] ) |
| Document  type | Article OR Review Article |
| Language | English |
| Timespan | 2005.01.01-2025.12.31 |
