# Supplementary material for: Application of multi-omics in systemic autoimmune rheumatic diseases: a bibliometric and visualization analysis
Source: Front Immunol. 2026 Apr 16;17:1759610. doi: 10.3389/fimmu.2026.1759610 (PMC13128600; doi:10.3389/fimmu.2026.1759610)
Supplement: Supplementary Table 2 — Top 20 globally cited publications and detailed information. [file SupplementaryFile2.docx]

| **Paper** | **DOI** | **Total Citations** | **TC per Year** | **Normalized TC** |
| --- | --- | --- | --- | --- |
| RAO DA, 2017, NATURE | 10.1038/nature20810 | 881 | 88.10 | 17.90 |
| TILLER T, 2008, J IMMUNOL METHODS | 10.1016/j.jim.2007.09.017 | 863 | 45.42 | 9.06 |
| ZHANG F, 2019, NAT IMMUNOL | 10.1038/s41590-019-0378-1 | 735 | 91.88 | 13.07 |
| NYGAARD G, 2020, NAT REV RHEUMATOL | 10.1038/s41584-020-0413-5 | 709 | 101.29 | 15.43 |
| MORSE C, 2019, EUR RESPIR J | 10.1183/13993003.02441-2018 | 551 | 68.88 | 9.80 |
| CHAUSSABEL D, 2008, IMMUNITY | 10.1016/j.immuni.2008.05.012 | 534 | 28.11 | 5.61 |
| ALIVERNINI S, 2020, NAT MED | 10.1038/s41591-020-0939-8 | 508 | 72.57 | 11.06 |
| TSUKUI T, 2020, NAT COMMUN | 10.1038/s41467-020-15647-5 | 490 | 70.00 | 10.67 |
| SOUYRIS M, 2018, SCI IMMUNOL | 10.1126/sciimmunol.aap8855 | 472 | 52.44 | 9.35 |
| CULEMANN S, 2019, NATURE | 10.1038/s41586-019-1471-1 | 454 | 56.75 | 8.07 |
| MIZOGUCHI F, 2018, NAT COMMUN | 10.1038/s41467-018-02892-y | 444 | 49.33 | 8.79 |
| MAUNAKEA AK, 2013, CELL RES | 10.1038/cr.2013.110 | 440 | 31.43 | 7.11 |
| TIPTON CM, 2015, NAT IMMUNOL | 10.1038/ni.3175 | 439 | 36.58 | 10.10 |
| ROYLE L, 2008, ANAL BIOCHEM | 10.1016/j.ab.2007.12.012 | 408 | 21.47 | 4.28 |
| DUTERTRE CA, 2019, IMMUNITY | 10.1016/j.immuni.2019.08.008 | 398 | 49.75 | 7.08 |
| KOMATSU N, 2022, NAT REV RHEUMATOL | 10.1038/s41584-022-00793-5 | 392 | 78.40 | 14.70 |
| WEI KV, 2020, NATURE | 10.1038/s41586-020-2222-z | 390 | 55.71 | 8.49 |
| NIE K, 2021, FRONT CELL INFECT MI | 10.3389/fcimb.2021.757718 | 386 | 64.33 | 10.97 |
| BERGHÖFER B, 2006, J IMMUNOL | 10.4049/jimmunol.177.4.2088 | 380 | 18.10 | 6.28 |
| TARDITO S, 2019, AUTOIMMUN REV | 10.1016/j.autrev.2019.102397 | 354 | 44.25 | 6.29 |
