# Supplementary material for: Application of multi-omics in systemic autoimmune rheumatic diseases: a bibliometric and visualization analysis
Source: Front Immunol. 2026 Apr 16;17:1759610. doi: 10.3389/fimmu.2026.1759610 (PMC13128600; doi:10.3389/fimmu.2026.1759610)
Supplement: Supplementary Table 4 — Analysis of selected randomized controlled trials (RCTs) identified from PubMed. [file SupplementaryFile4.docx]

**Tables4.PubMed Randomized Controlled Trial list**

| No. | First Author | Year | Title | Disease |
| --- | --- | --- | --- | --- |
| 1 | Baker T | 2024 | Type I interferon blockade with anifrolumab in patients with systemic lupus erythematosus modulates key immunopathological pathways in a gene expression and proteomic analysis of two phase 3 trials | SLE |
| 2 | Dörner T | 2024 | Efficacy and safety of remibrutinib, a selective potent oral BTK inhibitor, in Sjögren's syndrome | Sjögren's syndrome |
| 3 | Seridi L | 2021 | Novel signatures associated with systemic lupus erythematosus clinical response to IFN-α/-ω inhibition | SLE |
| 4 | Guo X | 2018 | Blockade of GM-CSF pathway induced sustained suppression of myeloid and T cell activities in rheumatoid arthritis | RA |
| 5 | Teitsma XM | 2018 | Explorative analyses of protein biomarkers in early rheumatoid arthritis achieving sustained drug-free remission | Early RA |
| 6 | Guo X | 2019 | Pharmacodynamic biomarkers and differential effects of TNF- and GM-CSF-targeting biologics in rheumatoid arthritis | RA |
| 7 | Teitsma XM | 2018 | Baseline metabolic profiles of early rheumatoid arthritis patients achieving sustained drug-free remission | Early RA |
| 8 | Agueusop I | 2025 | Deciphering differential biomarkers for anti-IL-6 receptor and anti-tumour necrosis factor-α treatment response in rheumatoid arthritis by multiomics analysis | RA |
